# Supplementary material for: Inference of Transmission Network Structure from HIV Phylogenetic Trees
Source: PLoS Comput Biol. 2017 Jan 13;13(1):e1005316. doi: 10.1371/journal.pcbi.1005316 (PMC5279806; doi:10.1371/journal.pcbi.1005316)
Supplement: S1 Text — (PDF) [file pcbi.1005316.s001.pdf]

Inference of transmission network structure from HIV phylogenetic trees

Federica Giardina[1,2*], Ethan Obie Romero-Severson[2], Jan Albert[3,4], Tom Britton[1], Thomas Leitner[2]

1 Department of Mathematics, Stockholm University, Stockholm, Sweden
2 Theoretical Biology and Biophysics Group, Los Alamos National Laboratory, Los Alamos, NM
3 Department of Microbiology, Tumor and Cell Biology, Karolinska Institute, Stockholm, Sweden
4 Department of Clinical Microbiology, Karolinska University Hospital, Stockholm, Sweden

*** E-mail: federica@math.su.se**

Supplementary information

Probability to escape infection

We calculated the probability to escape infection from a neighbour,π, as the exponential of the total infectious pressure. Let X denote the time to diagnosis (or death from AIDS if never diagnosed) and assume that X follows an exponential distribution with rate parameter γ, i.e. $X \sim Exp(\gamma)$. In the first model specification (constant infectivity) π can be written as $\pi = \mathbb{E}[Exp(-\lambda X)]$ where λ is the constant transmission rate. Therefore, $\pi = \mathbb{E}[Exp(-\lambda X)] = \int_0^{+\infty} \gamma e^{-\gamma x}(e^{-\lambda x})\mathrm{d}x = \frac{\gamma}{\gamma+\lambda}$.

In the second model specification, which included stage-varying infectivity, let t_1 denote the deterministic time spent in the first stage (acute phase), T_2 the random time spent in the second stage (chronic phase), represented by an exponential random variable with rate parameter β, i.e. $T_2 \sim Exp(\beta)$, and t_3 the time spent in the pre-AIDS stage. Here, the transmission rates in each of the three infection stages are λ_1, λ_2 and λ_3, respectively. Thus, the infectious pressure has a different expression depending on when the diagnosis occurs (in the acute, chronic or pre-AIDS stage). The probability π is the mean of the infectious pressure calculated over $X$and T_2. We have:
$\pi = \mathbb{E}[Exp(-\lambda_1 \mathbb{1}_{X<t_1} - (\lambda_1 t_1 + \lambda_2(X - t_1))\mathbb{1}_{t_1<X<t_1+T_2} - (\lambda_1 t_1 + \lambda_2 T_2 + \lambda_3(X - t_1 - T_2)\mathbb{1}_{X>t_1+T_2})] =$
$= \int_0^{t_1} \gamma e^{-\gamma x}(e^{-\lambda_1 x})\mathrm{d}x + \int_0^{\infty}\int_{t_1}^{t_1+t_2} \beta e^{-\beta t_2}\gamma e^{-\gamma x} e^{-(\lambda_1 t_1 + \lambda_2(x-t_1))}\mathrm{d}t_2\mathrm{d}x +$
$\int_0^{\infty}\int_{t_1+t_2}^{\infty} \beta e^{-\beta t_2}\gamma e^{-\gamma x} e^{-(\lambda_1 t_1 + \lambda_2 t_2 + \lambda_3(x-t_1-t_2))}\mathrm{d}t_2\mathrm{d}x$

$$= -\frac{\gamma}{\gamma+\lambda_1}(e^{-t_1(\gamma+\lambda_1)} - 1) + \frac{\gamma}{\beta+\gamma+\lambda_2}e^{-t_1(\gamma+\lambda_1)} + \frac{\beta}{\beta+\gamma+\lambda_2}\frac{\gamma}{\gamma+\lambda_3}e^{-t_1(\gamma+\lambda_1)}$$
$$= -\gamma e^{-t_1(\gamma+\lambda_1)}\left(\frac{1-e^{t_1(\gamma+\lambda_1)}}{\gamma+\lambda_1} - \frac{1}{\beta+\gamma+\lambda_2} - \frac{\beta}{(\beta+\gamma+\lambda_2)(\gamma+\lambda_3)}\right)$$

Equating the two expressions of π under the two different model specifications, we can calculate the three stage dependent transmission rates λ_1, λ_2 and λ_3 corresponding to a given λ. For example, let us assume:
$t_1 = 30, \beta = 1/(365 * 8), \gamma = 1/(2.8 * 365), \lambda_1 = 100\lambda_2, \lambda_3 = 100\lambda_2, \lambda = 0.001$. We obtain: $\lambda_2 = 0.000121$
