# Supplementary material for: Inference of Transmission Network Structure from HIV Phylogenetic Trees
Source: PLoS Comput Biol. 2017 Jan 13;13(1):e1005316. doi: 10.1371/journal.pcbi.1005316 (PMC5279806; doi:10.1371/journal.pcbi.1005316)
Supplement: S2 Text — (PDF) [file pcbi.1005316.s002.pdf]

Inference of transmission network structure from HIV phylogenetic trees

Federica Giardina[1,2*], Ethan Obie Romero-Severson[2], Jan Albert[3,4], Tom Britton[1], Thomas Leitner[2]

1 Department of Mathematics, Stockholm University, Stockholm, Sweden
2 Theoretical Biology and Biophysics Group, Los Alamos National Laboratory, Los Alamos, NM
3 Department of Microbiology, Tumor and Cell Biology, Karolinska Institute, Stockholm, Sweden
4 Department of Clinical Microbiology, Karolinska University Hospital, Stockholm, Sweden

*** E-mail: federica@math.su.se**

Supplementary information

Approximate Bayesian Computation for model choice

Within the ABC-SMC approach [1], particles are first generated from the prior distribution. Particles are then resampled from the obtained sample, and slightly perturbed. From these resampled particles, a new sample is formed, from which again particles are resampled, etc.

The threshold value ϵ for the summary statistic – below which new particles are accepted – is lowered with every newly obtained sample. As a result, the acceptance rate decreases and the acceptance threshold approaches zero with an increase in the number of iterations (resamplings).

Initial ϵ-values were estimated as follows. We generated 100 trees and we calculated the summary statistics (indices) and used the standard deviation of this distribution as the initial ϵ values. The ϵ-values were decreased in an exponential fashion as the sequential ABC scheme progresses.

- Initilize ϵ
- Set the population indicator $t = 1$
- Set the particle indicator $i = 1$
- If $t = 1$, sample (m'', θ'') from the prior $\pi(m, \theta) = \pi(m)\pi(\theta|m)$
- If $t > 1$ sample m' with probability $\pi_{t-1}(m')$ and perturb $m'' \sim Km_t(m|m')$ Sample θ' from the previous population $\{\theta(m'')_{t-1}\}$ with weights w_{t-1}. Perturb the particle, $\theta \sim KP_{t,m''}(\theta|\theta')$ where $KP_{t,m''}$ is the particle perturbation kernel. If $\pi(m'', \theta'') = 0$, repeat this step. Simulate a candidate dataset $x' \sim f(x|m'', \theta'')$ If $\rho(x', y) > \epsilon$ repeat this step.

- Set $(m_t^{(i)}, \theta_t^{(i)}) = (m'', \theta'')$ and $d_t^{(i)} = \rho(x', y)$, calculate the weight as $w_t^{(i)}(m_t^{(i)}, \theta_t^{(i)}) = \begin{cases} 1 & \text{if } t = 1 \\ \frac{\pi(m_t^{(i)}, \theta_t^{(i)})}{S_1 S_2} & \text{if } t > 0 \end{cases}$ where

$$S_1 = \sum_{j \in M} P_{t-1}(m_{t-1}^{(j)}) K m_t(m_t^{(i)} | m_{t-1}^{(j)}),$$

  and

$$S_2 = \sum_{k | m_t^{(i)} = m_{t-1}} \frac{w_{t-1}^{(k)} K P_{t, m_t^{(i)}}(\theta_t^{(i)} | \theta_{t-1}^{(k)})}{P_{t-1}(m_t^{(i)} = m_{t-1})}$$

- if $i < N$ set $i = i + 1$ and repeat the previous steps
- Normalize the weights. Obtain the marginal model probabilities given by

$$P_t(m_t = m) = \sum_{i | m_t^{(i)} = m_{t-1}} w_t^{(i)}(m_t^{(i)}, \theta_t^{(i)})$$

Implementation details

The algorithm was implemented in R [2] using the parallel package. The code is available by the authors under request. In the main manuscript ("ABC inference on transmission network type" in the Results section) we report the performance of the ABC-SMC algorithm on 100 simulated viral genealogies for each network type of size 1000. Here, in order to illustrate the scalability of the ABC-SMC algorithm and its computational cost, we repeated the same simulation study on 100 simulated viral genealogies for each network type of size 2000 and 3000.

The same parameters, prior distributions and tolerance levels were chosen for comparability. We record the number of times that the true model has the highest posterior model probability $P(M|D)$ among the three models for the 100 simulated datasets. The obtained posterior model probabilities were consistent with the case of networks of size 1000. Results are shown in Table 1 and Table 2 for networks of sizes 2000 and 3000, respectively.

We also report the computational time required on a parallel implementation on an IntelCore i7-4770S 3.10 GHZ 8-core processors (Table 3).

Table 1. Network type posterior probabilities. Networks of size 2000.

	WS	ER	BA
WS	**0.99**	0.01	0.00
ER	0.02	**0.77**	0.21
BA	0.01	0.22	**0.77**

Table 2. Network type posterior probabilities. Networks of size 3000.

	WS	ER	BA
WS	**0.98**	0.02	0.00
ER	0.03	**0.76**	0.21
BA	0.01	0.21	**0.78**

Table 3. Computational time.

	Network size		
	1000	2000	3000
Time	1.12h	1.50h	1.73h

References

1. Toni T, Welch D, Strelkowa N, Ipsen A, Stumpf MP. Approximate Bayesian computation scheme for parameter inference and model selection in dynamical systems. Journal of the Royal Society Interface. 2009;6(31):187–202.

2. R Core Team. R: A Language and Environment for Statistical Computing. Vienna, Austria; 2014. Available from: http://www.R-project.org/.
