# Supplementary figures and images for: Inference of Transmission Network Structure from HIV Phylogenetic Trees
Source: PLoS Comput Biol. 2017 Jan 13;13(1):e1005316. doi: 10.1371/journal.pcbi.1005316 (PMC5279806; doi:10.1371/journal.pcbi.1005316)

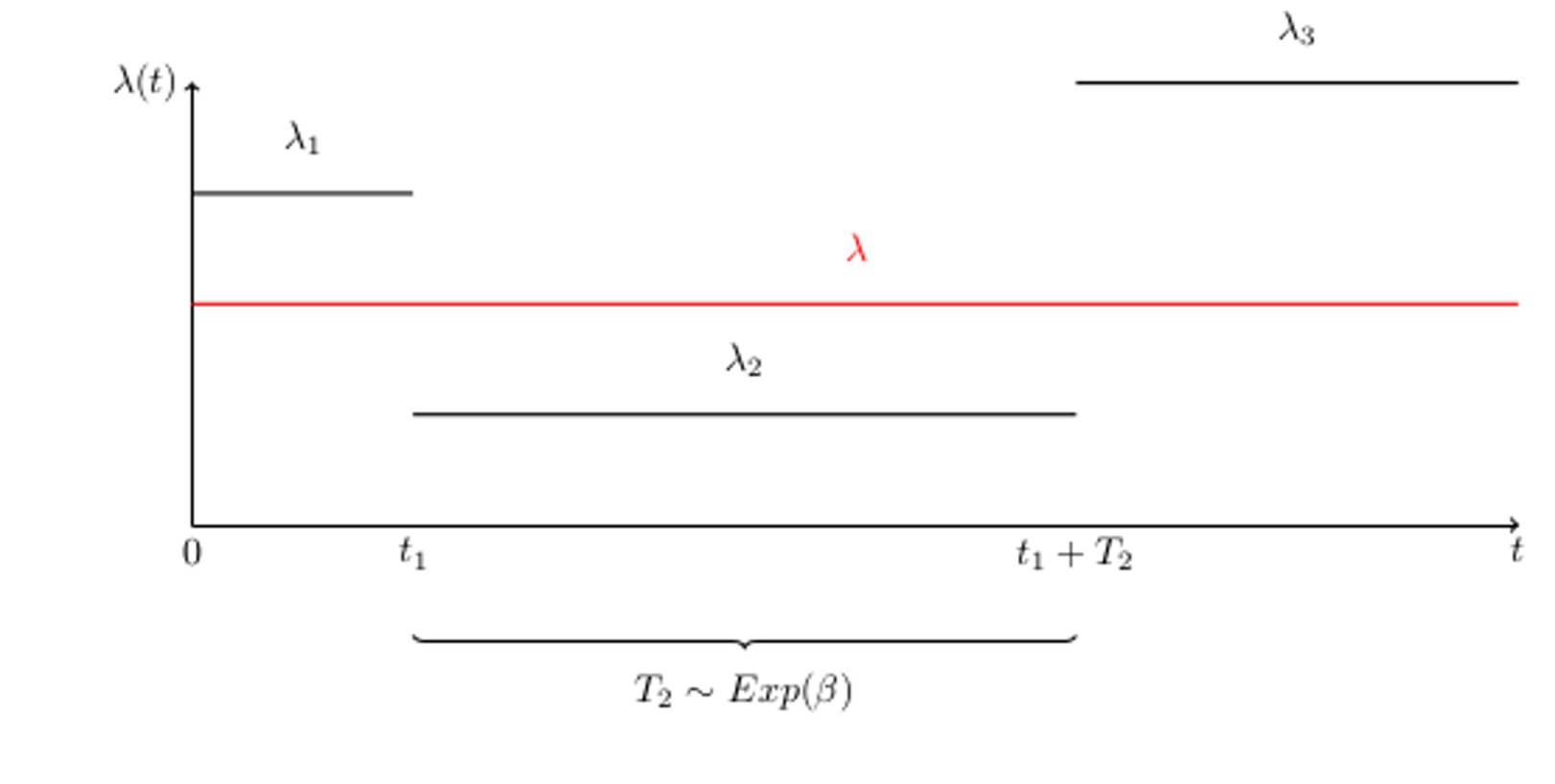

Supplement: S1 Fig — The first two model specifications represented: (i) constant rate (red line) and (ii) stage dependent infectivity (black lines). The length of the acute phase was assumed constant, t1 = 30 days while β was assumed to be 1/8 year−1. We do not assume a length for the AIDS phase a priori, but if an individual reaches the third stage, he will stay in the third phase until he is diagnosed or until death occurs. (TIF) [file pcbi.1005316.s003.tif]

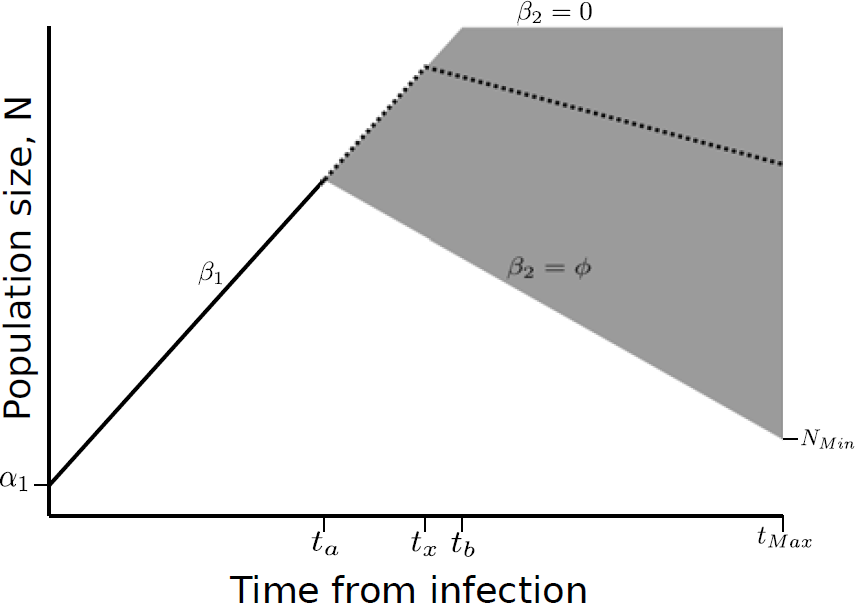

Supplement: S2 Fig — The effective viral population size is modelled as a two piece linear function: first, it grows at rate β1 until a random peak time tx, allowed to vary among individuals between ta and tb. After tx, the viral population size decreases or stabilizes at a rate β2. The dashed line represents one possible realization. This figure is part of [21]. (TIF) [file pcbi.1005316.s004.tif]

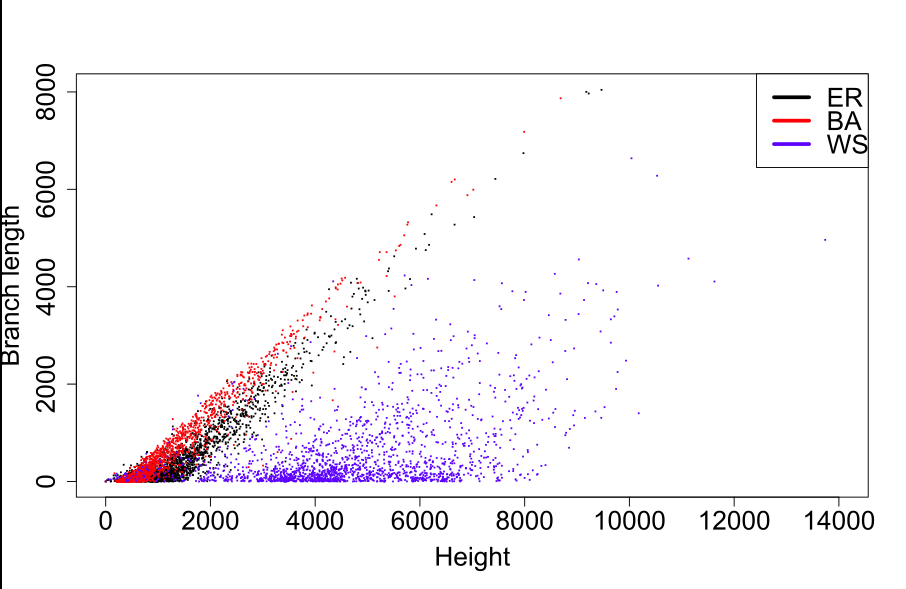

Supplement: S3 Fig — Mean branch length is evaluated at each coalescence event (originating internal branches) and each sampling event (external branches) until every individual is sampled. WS (red), ER (green), BA (blue). (TIF) [file pcbi.1005316.s005.tif]

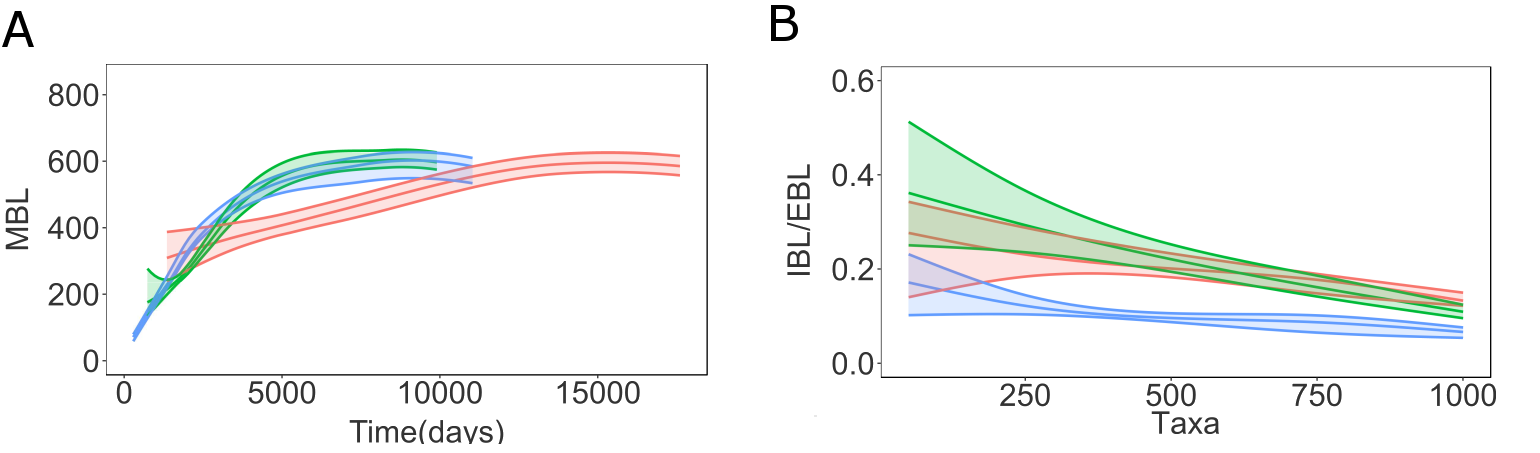

Supplement: S4 Fig — Mean branch length (MBL) as function of tree height (A) and internal/external branch length ratio (B) as function of the number of taxa for simulated outbreaks on networks of size 1000. The envelopes represent 95% confidence intervals around the medians. The curves are obtained using local regression (LOESS). WS (red), ER (green), BA (blue). (TIF) [file pcbi.1005316.s006.tif]

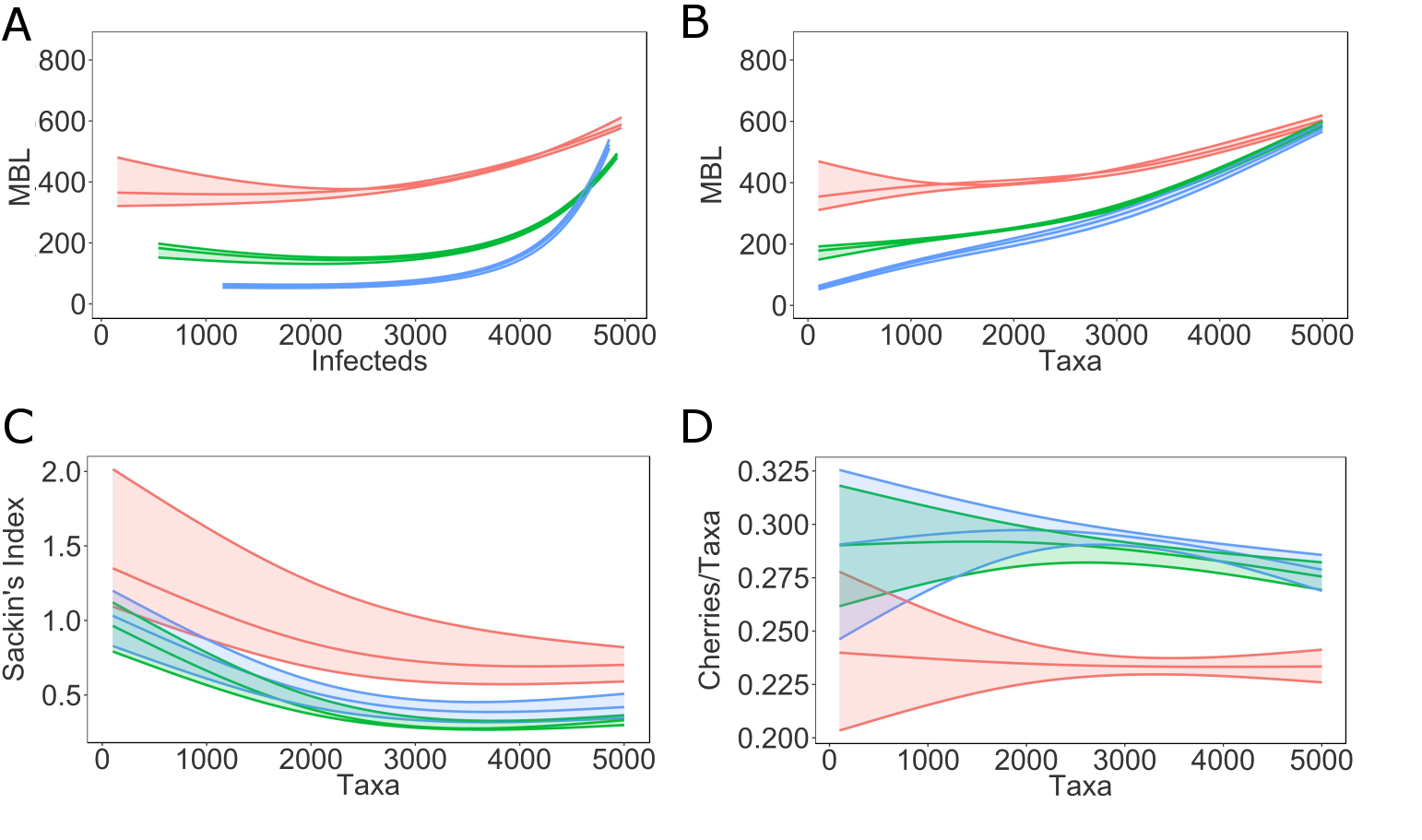

Supplement: S5 Fig — Mean branch length (MBL) as function of the number of infected individuals (A) and as function of the number of taxa (sampled infected individuals) (B) for simulated outbreaks on networks of size 5000 as epidemics progress. Note that there is a time interval between infections and diagnoses (which correspond to removal/sampling times). Sackin’s index (C) and number of cherries per taxa (D) as function of the number of taxa in networks of size 1000. The envelopes represent 95% confidence intervals around the medians. The curves are obtained using local regression (LOESS). WS (red), ER (green), BA (blue). (TIF) [file pcbi.1005316.s007.tif]

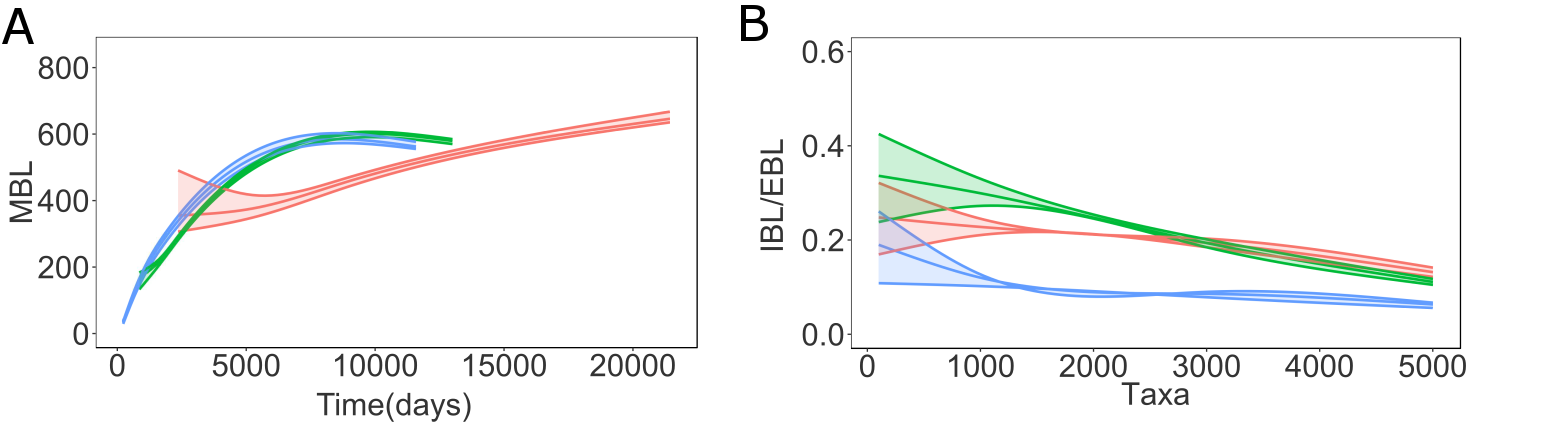

Supplement: S6 Fig — Mean branch length (MBL) as function of tree height (A) and internal/external branch length ratio (B) as function of the number of taxa for simulated outbreaks on networks of size 5000. The envelopes represent 95% confidence intervals around the medians. The curves are obtained using local regression (LOESS). WS (red), ER (green), BA (blue). (TIF) [file pcbi.1005316.s008.tif]

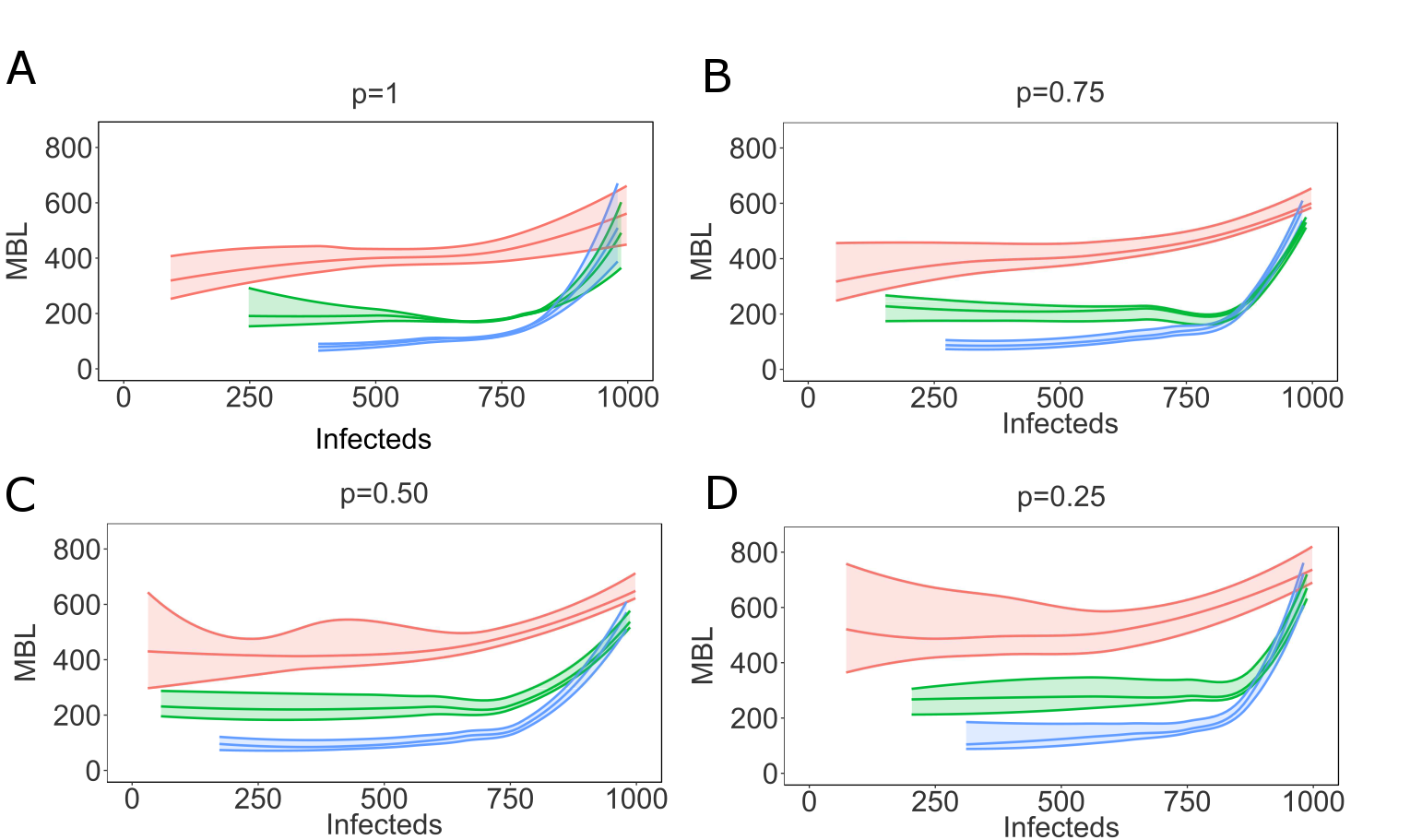

Supplement: S7 Fig — The mean branch length as function of number of infecteds, with varying sampling fraction (p = 1-0.25). The envelopes represent 95% confidence intervals around the medians. The curves are obtained using local regression (LOESS). WS (red), ER (green), BA (blue). (TIF) [file pcbi.1005316.s009.tif]
