# Supplementary material for: Inference of Transmission Network Structure from HIV Phylogenetic Trees
Source: PLoS Comput Biol. 2017 Jan 13;13(1):e1005316. doi: 10.1371/journal.pcbi.1005316 (PMC5279806; doi:10.1371/journal.pcbi.1005316)
Supplement: S1 Table — (PDF) [file pcbi.1005316.s010.pdf]

Inference of transmission network structure from HIV phylogenetic trees

Federica Giardina[1,2*], Ethan Obie Romero-Severson[2], Jan Albert[3,4], Tom Britton[1], Thomas Leitner[2]

1 Department of Mathematics, Stockholm University, Stockholm, Sweden
2 Theoretical Biology and Biophysics Group, Los Alamos National Laboratory, Los Alamos, NM
3 Department of Microbiology, Tumor and Cell Biology, Karolinska Institute, Stockholm, Sweden
4 Department of Clinical Microbiology, Karolinska University Hospital, Stockholm, Sweden

*** E-mail: federica@math.su.se**

Supplementary information

S1 Tab. Effect of increased rewiring probability (ρ) on network identification.

$\rho = 0.01$	WS	ER	BA
WS	0.99	0.01	0.00
ER	0.03	0.76	0.21
BA	0.01	0.21	0.78

$\rho = 0.1$	WS	ER	BA
WS	0.95	0.05	0.00
ER	0.11	0.70	0.19
BA	0.01	0.19	0.80

$\rho = 0.25$	WS	ER	BA
WS	0.75	0.16	0.09
ER	0.16	0.63	0.21
BA	0.06	0.15	0.79

$\rho = 0.5$	WS	ER	BA
WS	0.65	0.17	0.18
ER	0.19	0.57	0.24
BA	0.10	0.21	0.69
